# Supplementary material for: The Arabidopsis thaliana–Streptomyces Interaction Is Controlled by the Metabolic Status of the Holobiont
Source: Int J Mol Sci. 2022 Oct 26;23(21):12952. doi: 10.3390/ijms232112952 (PMC9655247; doi:10.3390/ijms232112952)
Supplement: Supplementary file 1 [file ijms-23-12952-s001.zip › Suplementary Materials.pdf]

Supplementary Figures and Tables

Figure S1. Characteristics of *A. thaliana* wild-type and *chs5* plants used for metabolomic profiling. Pictures of the mutants compared to wild-type (WT) plants: the chlorotic phenotype was visible when plants were grown under LTC.

Figure S2. Disease symptoms of the wild-type (WT) or *chs5* plants infected by Pto DC3000. Leaves were syringe-infiltrated with 5 x 10^5 CFU/mL of Pst DC3000 and photography were taken six days after inoculation. The whole plant is shown on the left. Diseased leaves are shown on the right.

Figure S3. Infection of wild type and *chs5* by *P. syringae* pv. *tomato* DC3000 (Pto DC3000). (**a**) Pathogen numerations were done for the wild type (WT) and the mutant *chs5*, at 6dpi. 3 independent experiments were performed with 6-8 week-old plants. N: number of replicates. The first infection was proceeded with N=7 for WT (average rosette diameter 7.2 cm, average number of leaves 19) and N=9 for *chs5* (average rosette diameter 6.8 cm, average number of leaves 18). Infection 2 was proceeded with N=8 (average rosette diameter 8.12 cm for WT and 7.2 cm for *chs5*, average number of leaves 18.9 for WT and 18.1 for *chs5*). Last infection was proceeded with N=12 (average rosette diameter 7.6 cm for WT and 6.6 cm for *chs5*, average number of leaves 20.4 for WT and 19.1 for *chs5*). There is a significant enhancement of *chs5* susceptibility compared to the WT. (p = p-value, one-way ANOVA test). (**b**) Stigmasterol content was quantified 6 days post-infection, by GC-MS. The y-axis corresponds to the ratio between the pic height and the sample dry weight. There is a significant enhancement of stigmasterol in infected WT or *chs5*, compared to non-infected plants (NI). (p = p-value, non-parametric Kruskal-Wallis test, the p-value were evaluated with a Dunn test and the Bonferroni correction).

Figure S4. Comparison of the total and the relative abundance (%) of the different phyla, class, order and family identified in the wild type (WT) or *chs5* mutant. (**a**) grown in STC at two different growth stages. (**b**) grown in STC conditions at the rosette stage. For this analysis, we compared 1 biological replicates with 3 technical replicates in each case. (**c**) grown in LTC at the silique stage. The relative abundance of each phylum, class, order or family in the total microbiota (all compartments) is expressed after down-sampling.

Figure S5. α- and β-diversity analysis revealed differences and similarities in wild-type (WT) and *chs5* mutant community structures, in three compartments, at both silique and rosette stages,

when grown in STC. (**a**) Comparison of α-diversity of the wild-type and *chs5* and in the three compartments (phyllosphere, rhizosphere and roots). Four different α-diversity indexes were used to study the global richness and diversity, in the different compartments (see material and methods): the richness was evaluated using the "observed" or the Chao1 indexes, the evenness (representing the phylogenetic diversity) was expressed with the Shannon and the Inverse Simpson indexes. The richness and evenness are higher in the rhizosphere and roots than phyllosphere when grown in STC. No significant difference was observed between WT and *chs5* (ANOVA, *p*-value = 0.954, 0.962, 0.396, 0.187 for Observed, Chao1, Shanon and InvSimpson indexes, respectively). Clear colors: *chs5*; deep colors: WT. (**b**) β-diversity analysis illustrated by clustering trees. Four β-diversity indexes were used to compare the different samples (see material and methods). The Bray-Curtis and the Jaccard indexes allowed to compare the composition of the communities, whereas the Unifrac or weighted Unifrac allowed to compare the phylogenetic diversity [56]. (**c**) MultiDimensional Scaling (MDS/PCoA) based on these indexes, the community composition and the phylogenetic diversity were distinct in the three plant compartments when grown STC. The ellipses were drawn at the 95% confidence interval of standard error and the mean value of the groups. R1, R2 and R3 correspond to three independent replicates.

Figure S6. α- and β-diversity analysis revealed differences and similarities in wild-type (WT) and mutant *chs5* community structures, in three compartments, at silique when grown in LTC. (**a**) Comparison of α-diversity of the wild-type and *chs5* and in the three compartments (phyllosphere, rhizosphere and roots). Four different α-diversity indexes were used to study the global richness and diversity, in the different compartments (see material and methods): the richness was evaluated using the "observed" or the Chao1 indexes, the evenness (representing the phylogenetic diversity) was expressed with the Shannon and the Inverse Simpson indexes. The richness and evenness are higher in the rhizosphere and roots than phyllosphere, when grown in LTC. Significant difference was observed between WT and *chs5* for observed, Shannon and invSipson indexes (ANOVA, *p*-value = 0.0429, 0.12, 0.0201, 0.0206 for Observed, Chao1, Shanon and InvSimpson indexes, respectively). Clear colors: *chs5*; deep color: WT. (**b**) β-diversity analysis illustrated by clustering trees. Four β-diversity indexes were used to compare the different samples (see material and methods). The Bray-Curtis and the Jaccard indexes allowed to compare the composition of the communities, whereas the Unifrac or weighted Unifrac allowed to compare the phylogenetic diversity [56]. (**c**) MultiDimensional Scaling (MDS/PCoA) based on these indexes, the community composition and the phylogenetic diversity were

slightly distinct in the three plant compartments, when grown in LTC. The ellipses were drawn at the 95% confidence interval of standard error and the mean value of the groups. R1, R2 and R3 correspond to three independent replicates.

Figure S7. Comparison of the relative phyla abundance in each compartment. The relative abundance was calculated in total or in variable microbiota, in the *chs5* mutant as compared to the wild type, at the rosette stage, when grown in STC. The relative abundance of each phylum is expressed as a percentage of the total microbiota (left) or as percentage of the total number of OTUs whose abundance is different in the wild type and in the mutant (variable, right).

Figure S8. Comparison of the community structures at both silique and rosette stages based on α- and β-diversity analysis revealed differences and similarities in the wild type and the mutant grown in STC. (**a**) Comparison of α-diversity of the wild-type (WT) and *chs5* at both stages (rosette and silique, called stem). Four different α-diversity indexes were used to study the global richness and diversity, in the different compartments (see material and methods): the richness was evaluated using the "observed" or the Chao1 indexes, the evenness (representing the phylogenetic diversity) was expressed with the Shannon and the Inverse Simpson indexes. When grown in STC, the richness and evenness are similar at both stages. No significant difference was observed between WT and *chs5* (ANOVA, *p*-value = 0.973, 0.9772, 0.649, 0.376 for Observed, Chao1, Shanon and InvSimpson indexes, respectively). Clear colors: *chs5*; deep colors: WT. (**b**) β-diversity analysis illustrated by clustering trees. Four β-diversity indexes were used to compare the different samples (see material and methods). The Bray-Curtis and the Jaccard indexes allowed to compare the composition of the communities, whereas the Unifrac or weighted Unifrac allowed to compare the phylogenetic diversity [56]. (**c**) MultiDimensional Scaling (MDS/PCoA) based on these indexes, the community composition and the phylogenetic diversity were distinct at the rosette and silique stages, when grown in STC. The ellipses were drawn at the 95% confidence interval of standard error and the mean value of the groups. R1, R2 and R3 correspond to three independent replicates.

Figure S9. Comparison of the relative phyla abundance in total or in variable microbiota of plants grown in STC at the silique or at the rosette stage, in the *chs5* mutant or in the wild type. The relative abundance of each phylum is expressed as a percentage of the total microbiota (first histogram) or as percentage of the total number of OTUs whose abundance is different at the silique and rosette stages.

Figure S10. BioNJ phylogenetic tree of the OTUs identified in this study. This tree includes the sequences deposited in public databases which show greater similarities with the identified OTUs. This phylogenic tree was constructed using the "A la carte" options available on the phylogeny platform (http://www.phylogeny.fr/). Accession numbers are included in brackets in the figure.

Figure S11. Strategy used to separate phyllosphere, roots and rhizosphere, and to analyze bacterial community inhabiting these compartments. (a) The wild-type and *chs5* plants were extracted from soil at the two stages according to the number of leaves, diameter of the rosettes and length of the stems (see **Figure 1**). Plants were shaken, then the plants were cut to separate phyllosphere and roots/rhizosphere. Rhizosphere (adherent soil) was separated from roots by scrapping them using a sterile scraper, and roots shortly washed in sterile distilled water to remove soil. **(b)** DNA was extracted from these samples, 16S rRNA encoding gene were amplified and sequenced using the Miseq Illumina Technology. Two pipelines were used to analyze sequences (FROGS and Phyloseq, see materials and methods).

Figure S12. Primers used for library generation. (a) The PCR1 primers contain: - the 16S V5-V6 specific sequence, - the 0 to 7 pb heterogeneity spacer (0-7 bp HS), - a part of the Read 1 or Read 2 specific sequences used for sequencing primer hybridization (Rd1 SP or Rd2 SP). The PCR2 primers contain: - a part of the Read 1 or Read 2 specific sequences used for sequencing primer hybridization (Rd1 SP or Rd2 SP), an index (Nextera XT N5XX or S7XX), a P5 or P7 sequence used to hybridize the libraries on the flowcell and for cluster generation. **(b)** example of a PCR amplicon profile obtained using these primers and one DNA extraction mixture as matrix and compared as a negative control. In the samples, two sizes of products were obtained corresponding to bacterial or plastidial (≈ 600 nucleotides) and mitochondrial (≈ 1000 nucleotides) 16S rRNA encoding gene.

Figure S13. Rarefaction Curve obtained from NGS sequencing data. (**a**) The wild type (WT) versus *chs5* grown in LTC. (**b**) The wild type (WT)versus *chs5* grown in STC, at the rosette stage (first experiment) and silique stage. (**c**) The wild type versus *chs5* grown in STC, at the rosette stage (second experiment). These curves are plots of the number of classes or genera as a function of the number of samples. R1, R2 and R3 correspond to three independent replicates.

Supplementary Tables

Table S1: Metabolites differentially accumulated in *chs5* mutant as compared to the wild type, when grown in LTC.

Table S2: Metabolites differentially accumulated in *chs5* mutant as compared to the wild type, when grown in STC.

Table S3: Metabolites differentially accumulated in *chs5* mutant as compared to the wild type, in both LTC and STC. Only metabolites with *p*-value <0.05 and a level 2 or 3 annotation according to Schymanski [48] are given.

Table S4: OTUs (=cluster) whose abundance is different at the rosette stage between the wild-type and the *chs5* plants, grown in STC in the phyllosphere (**a**), the rhizosphere (**b**) or the root (**c**) compartments. Positive value of the log2-fold change revealed that the OTU is more abundant in the wild-type than the mutant plants. Negative value of the log2-fold change revealed that the OTU is more abundant in the mutant than the wild-type plants.

Table S5: OTUs (=cluster) whose abundance is different at the silique stage between the wild-type and the *chs5* plants, grown in STC. Positive value of the log2-fold change revealed that the OTU is more abundant in the wild-type than the mutant plants. Negative value of the log2-fold change revealed that the OTU is more abundant in the mutant than the wild-type plants.

Table S6: OTUs (=cluster) whose abundance is different between the wild-type and the *chs5* plants, grown in LTC in the phyllosphere (**a**), the rhizosphere (**b**) or the root (**c**) compartments. Positive value of the log2-fold change revealed that the OTU is more abundant in the wild-type than the mutant plants. Negative value of the log2-fold change revealed that the OTU is more abundant in the mutant than the wild-type plants.

Table S7: OTUs (=cluster) with variable abundance (*p*-value<0.01) between rosette and silique stage, in the wild type (**a**) and in *chs5* (**b**). Positive value of the log2-fold change revealed that the OTU is less abundant at the rosette than the silique stage. Negative value of the log2-fold change revealed that the OTU is more abundant at the rosette than the silique stage.

Table S8: Comparison of the 16S rRNA sequences obtained using the metabarcoding and the gene capture approaches, and with sequences found in nucleotide collection (nr/nt) (https://blast.ncbi.nlm.nih.gov, analyzed the 6 October 2020).

Table S9: Primers used for 16S rRNA encoding gene amplification.

References

48. Schymanski, E.L.; Jeon, J.; Gulde, R.; Fenner, K.; Ruff, M.; Singer, H.P.; Hollender, J. Identifying Small Molecules via High Resolution Mass Spectrometry: Communicating Confidence. *Environ. Sci. Technol.* **2014**, *48*, 2097–2098, doi:10.1021/es5002105.

56. McMurdie, P.J.; Holmes, S. Phyloseq: An R Package for Reproducible Interactive Analysis and Graphics of Microbiome Census Data. *PLoS ONE* **2013**, *8*, e61217, doi:10.1371/journal.pone.0061217.

Figure S1

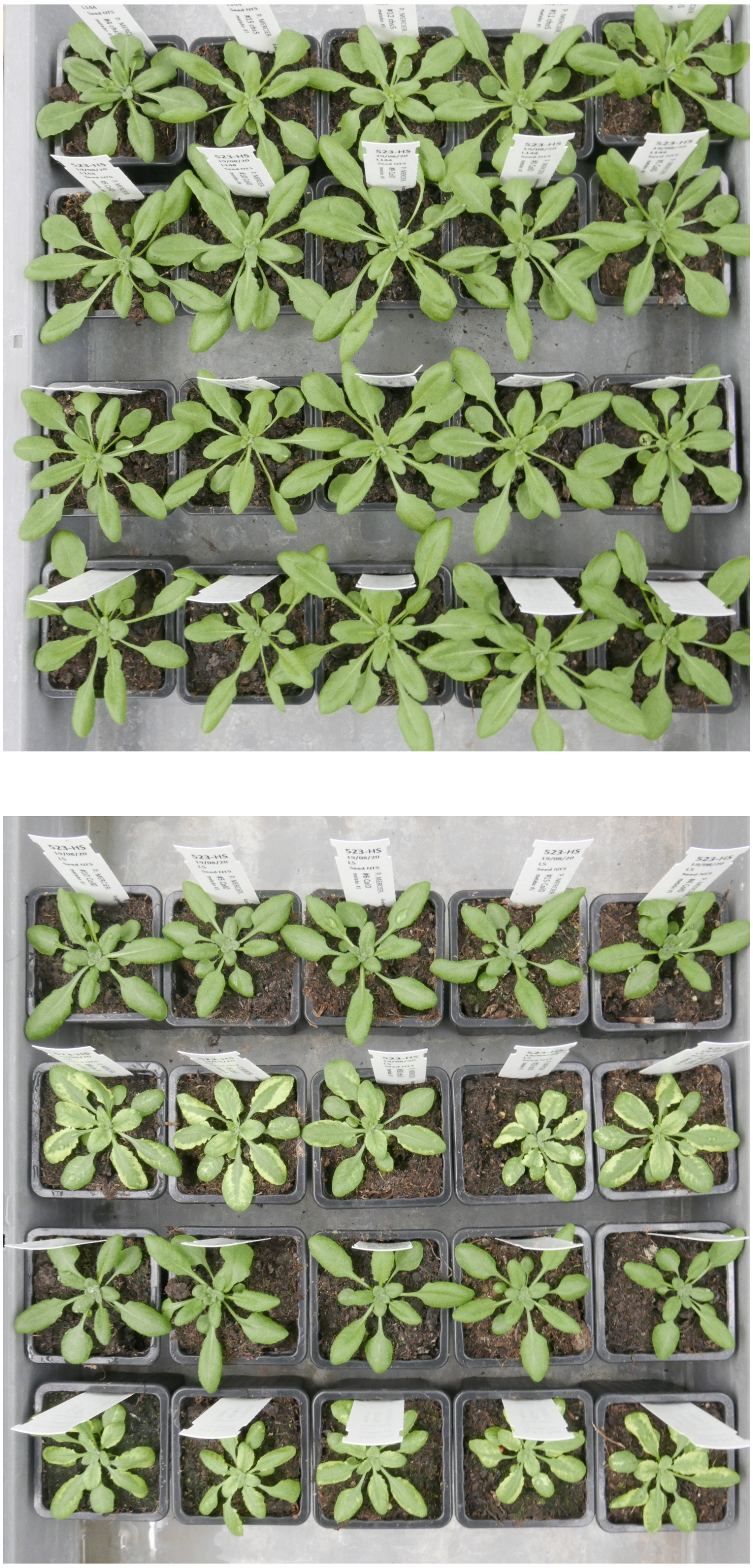

Figure S2

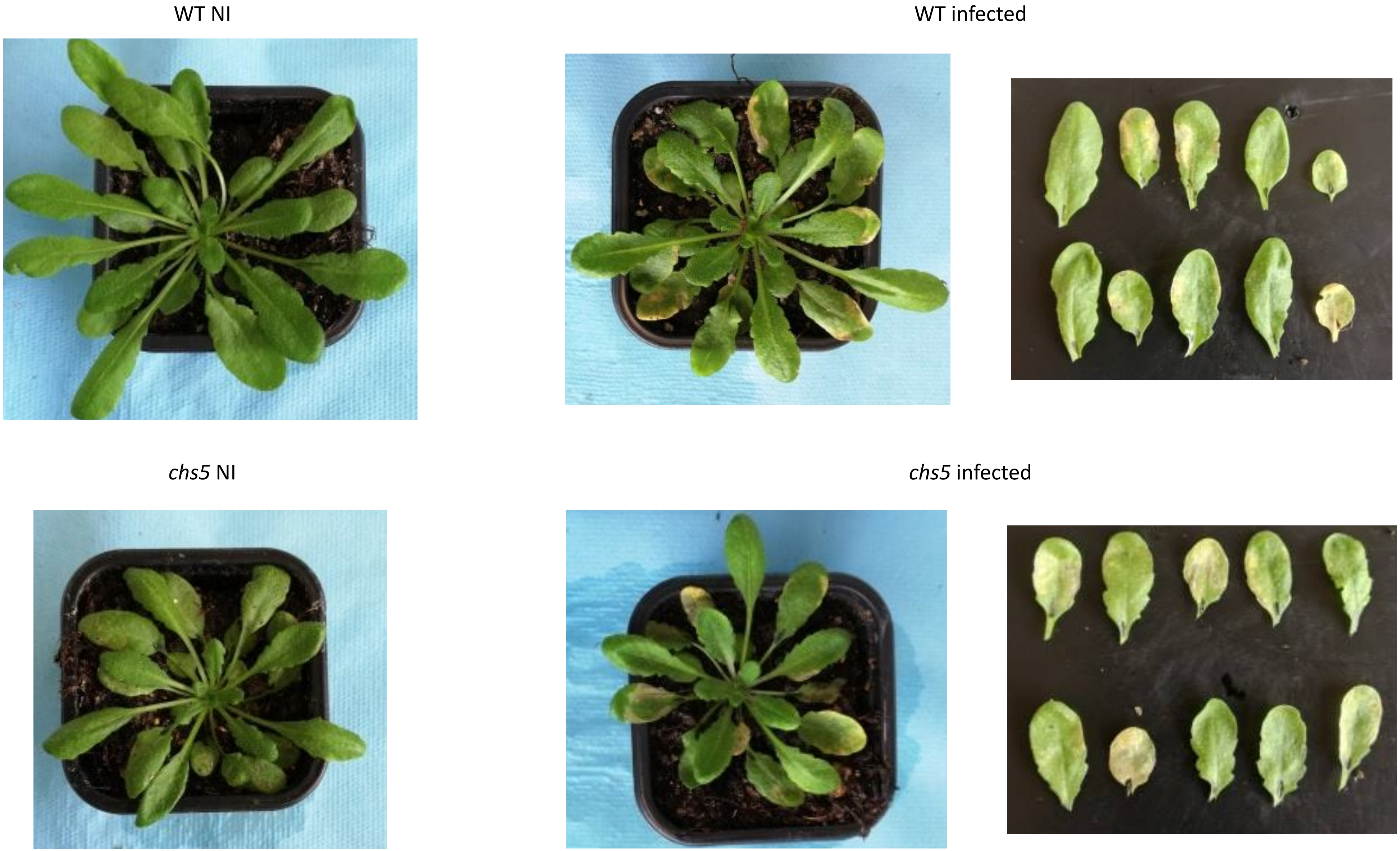

Figure S3

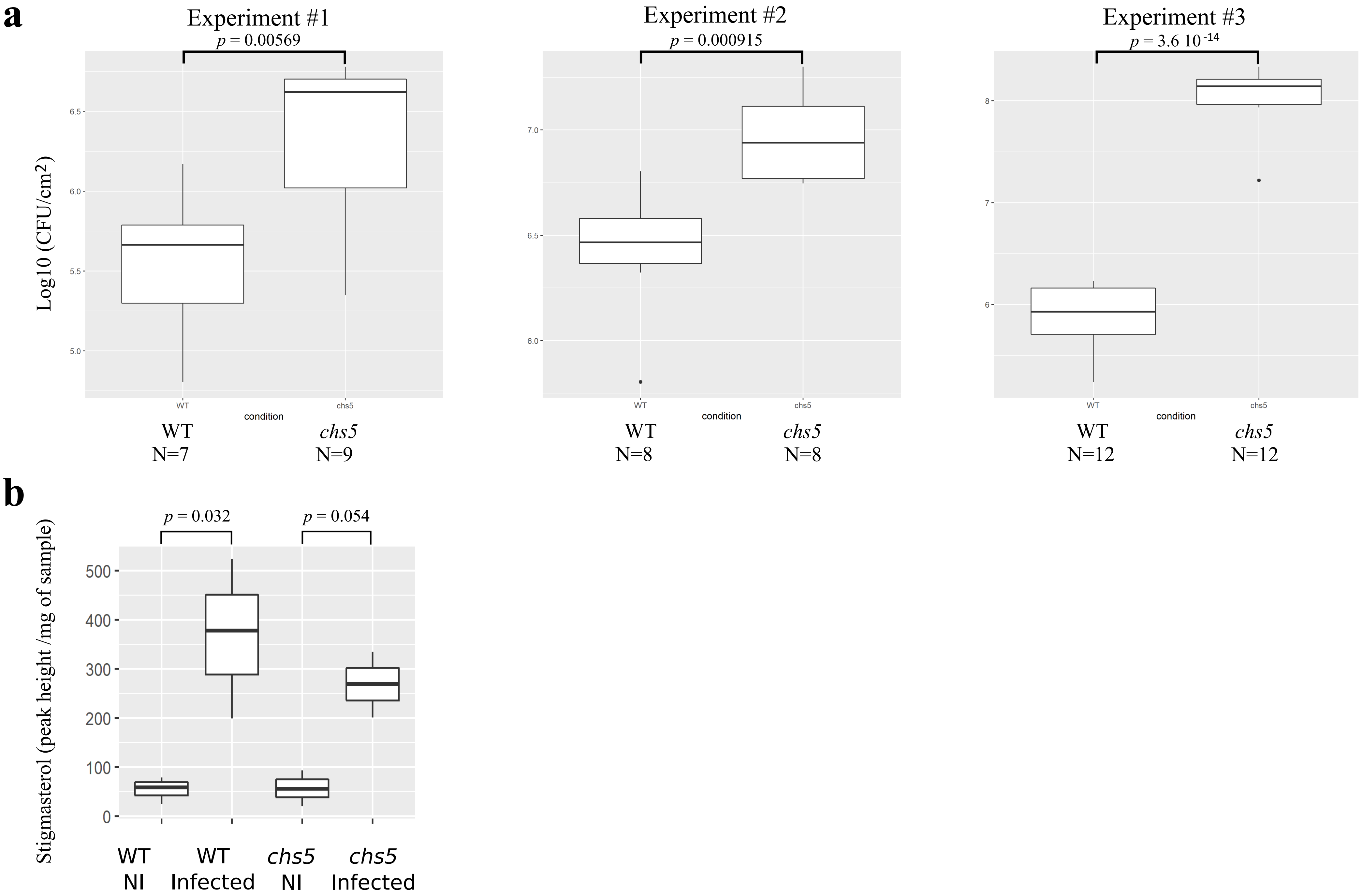

a
Experiment #1
p = 0.00569
Log10 (CFU/cm2)
6.5
6.0
5.5
5.0
WT
chs5
condition
WT
N=7
chs5
N=9
Experiment #2
p = 0.000915
7.0
6.5
6.0
WT
chs5
condition
WT
N=8
chs5
N=8
Experiment #3
p = 3.6 10 -14
8
7
6
WT
chs5
condition
WT
N=12
chs5
N=12
b
p = 0.032
p = 0.054
Stigmasterol (peak height /mg of sample)
500
400
300
200
100
0
WT
NI
WT
Infected
chs5
NI
chs5
Infected

Figure S4

a

Total abundance

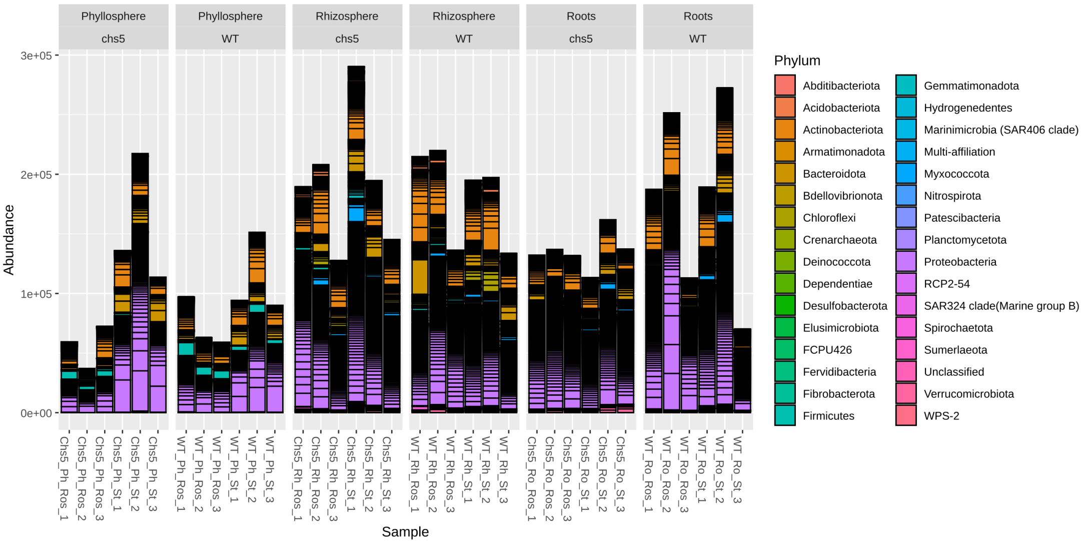

Relative abundance

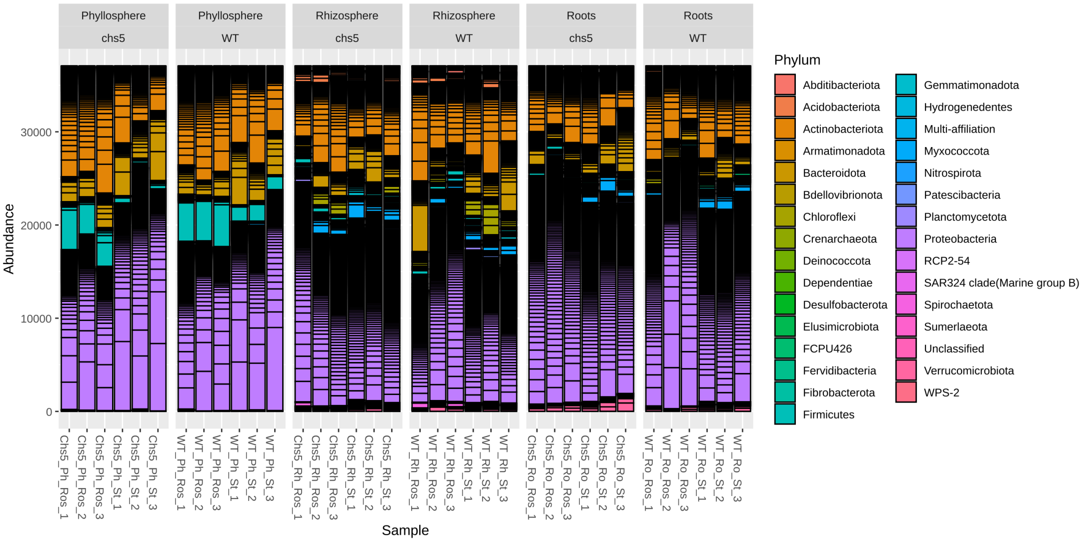

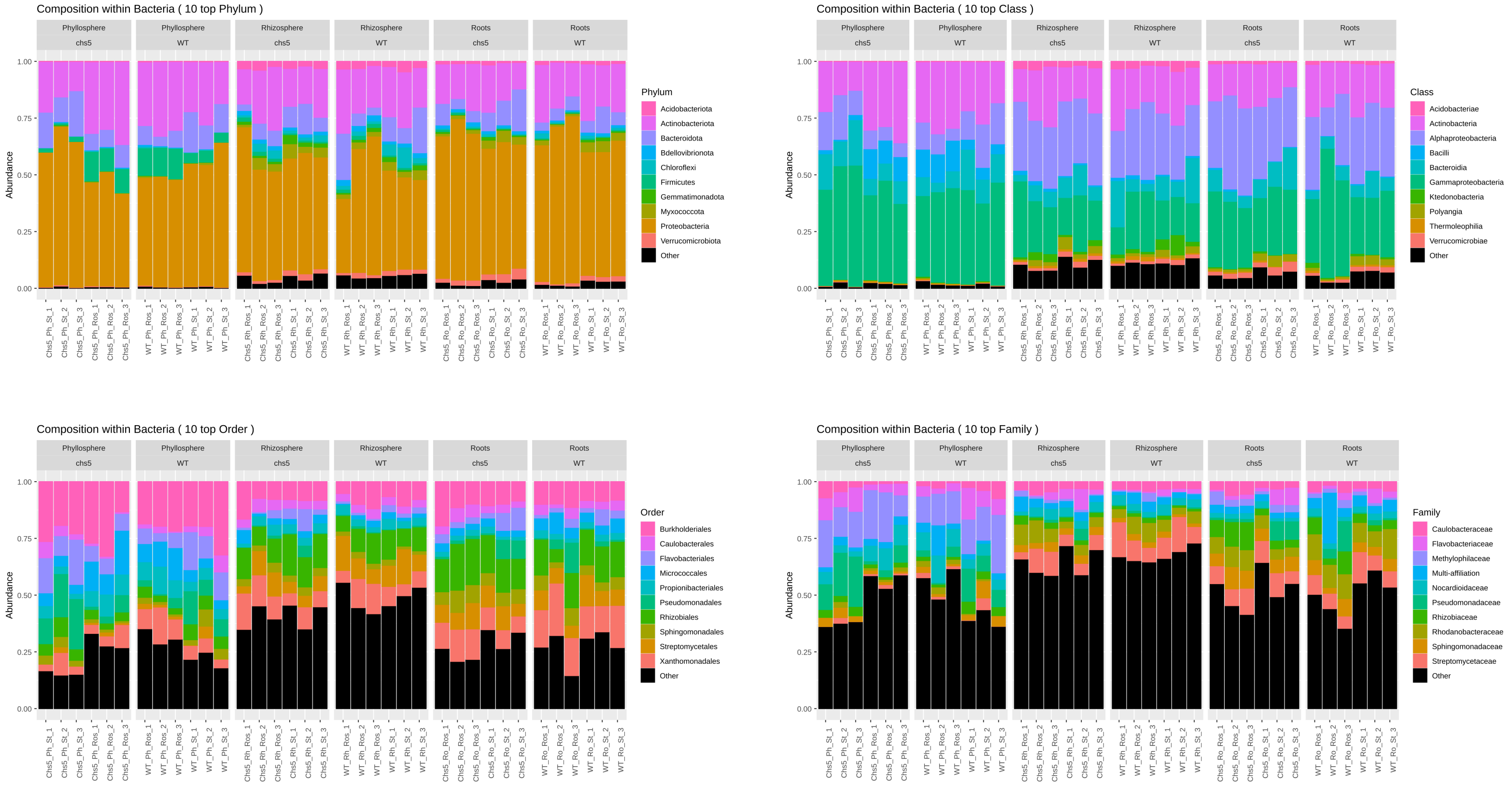

b

Total abundance

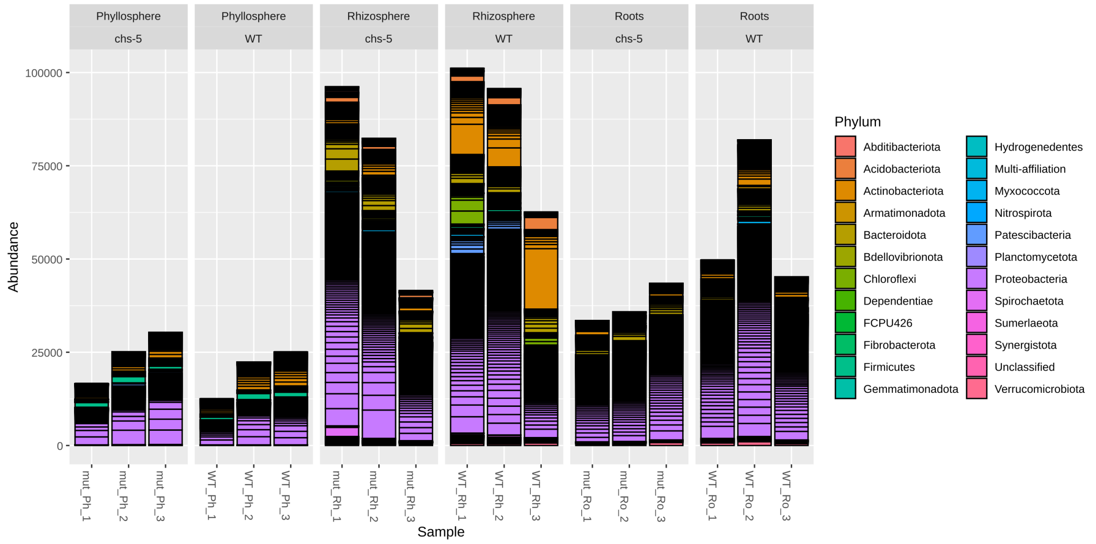

Relative abundance

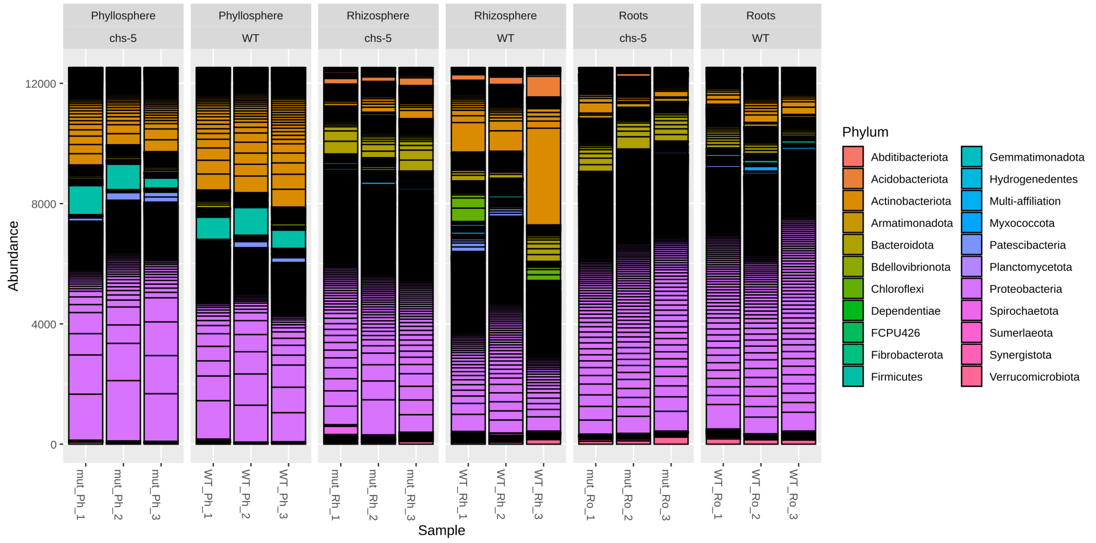

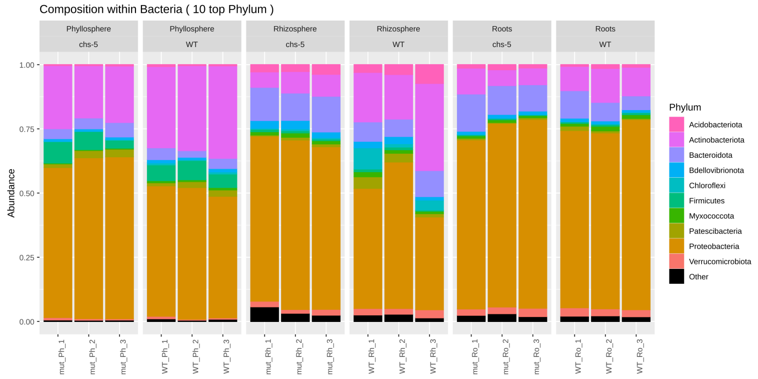

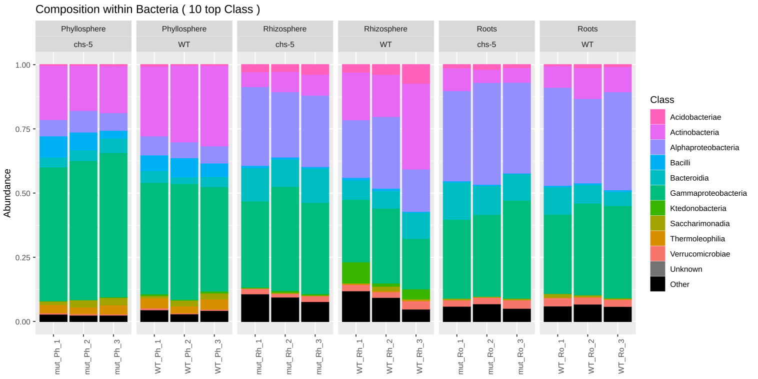

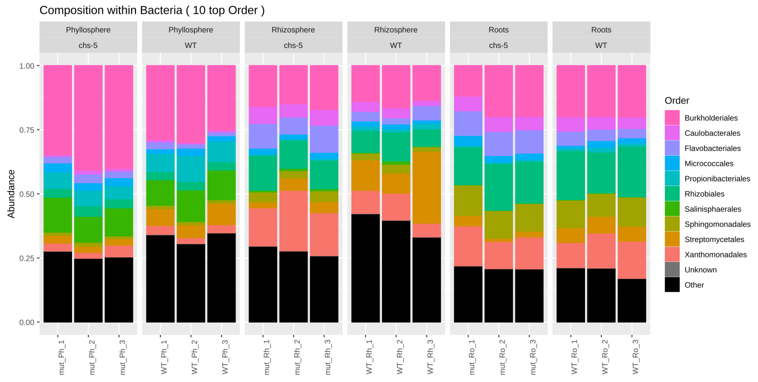

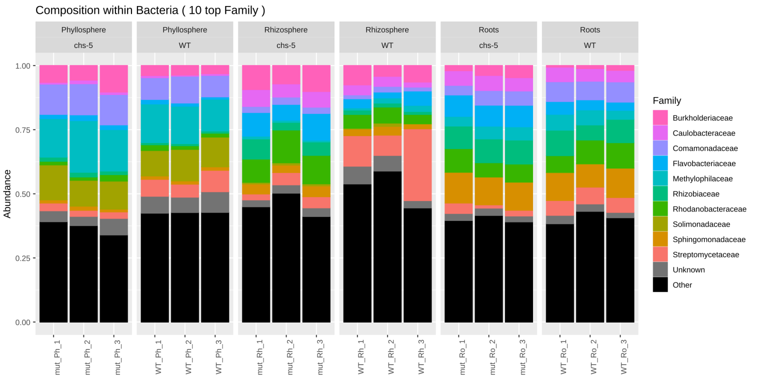

c

Total abundance

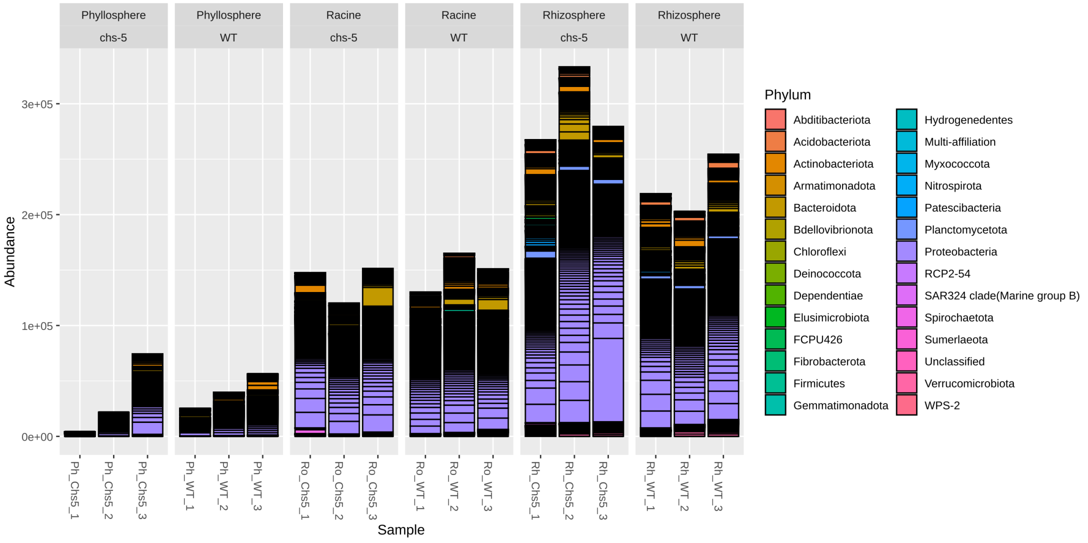

Relative abundance

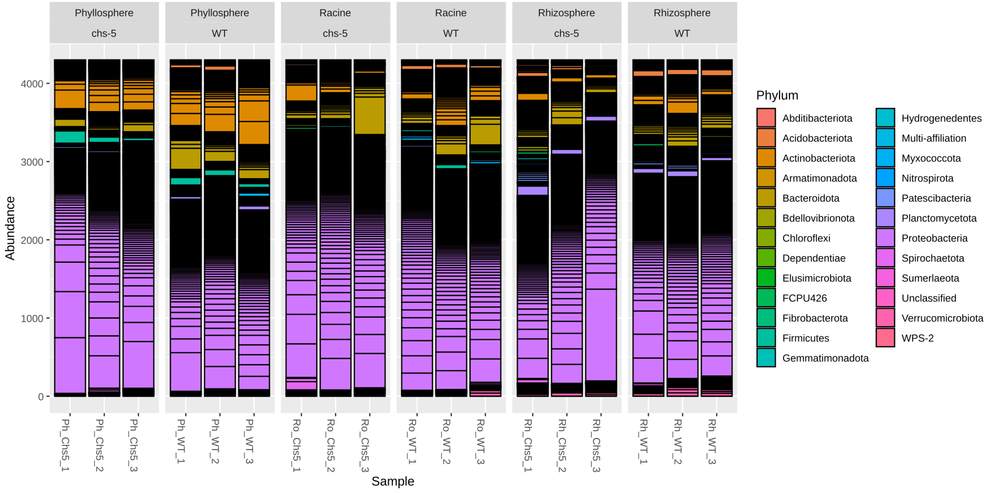

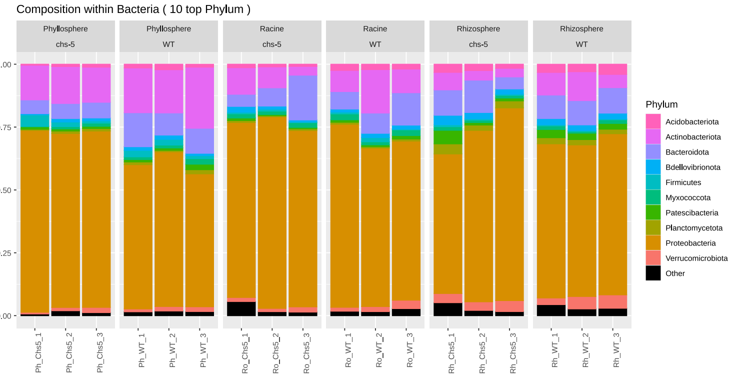

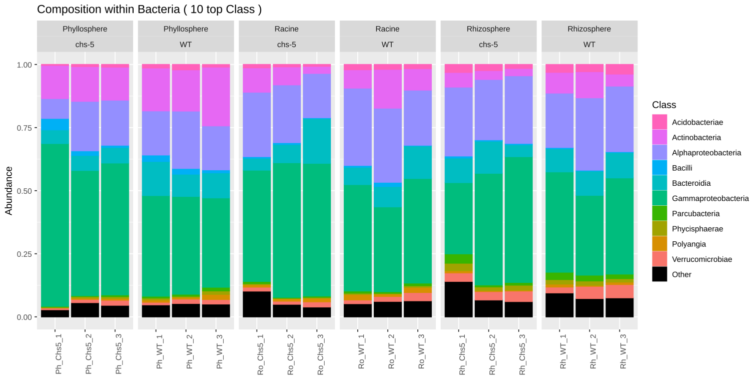

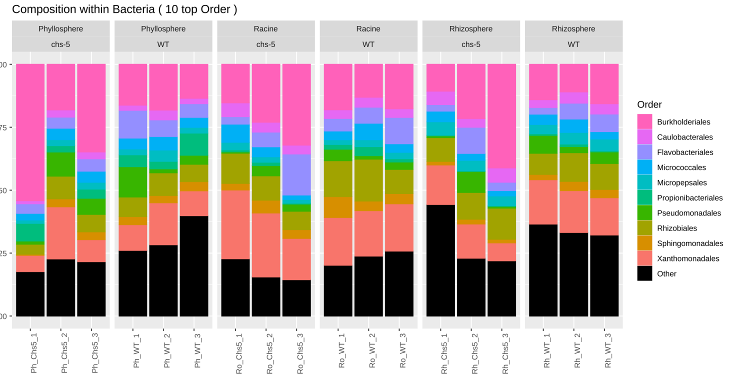

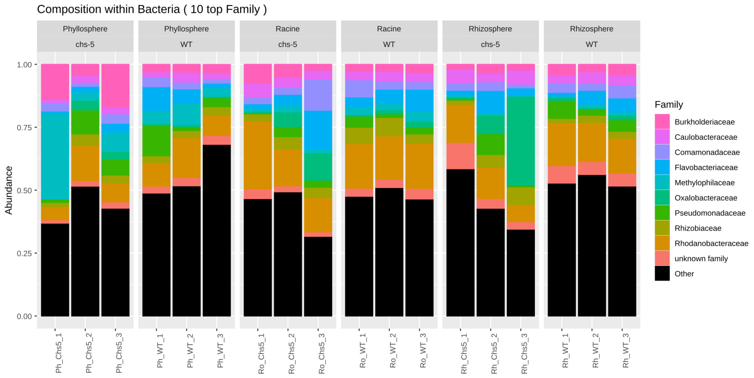

Figure S5

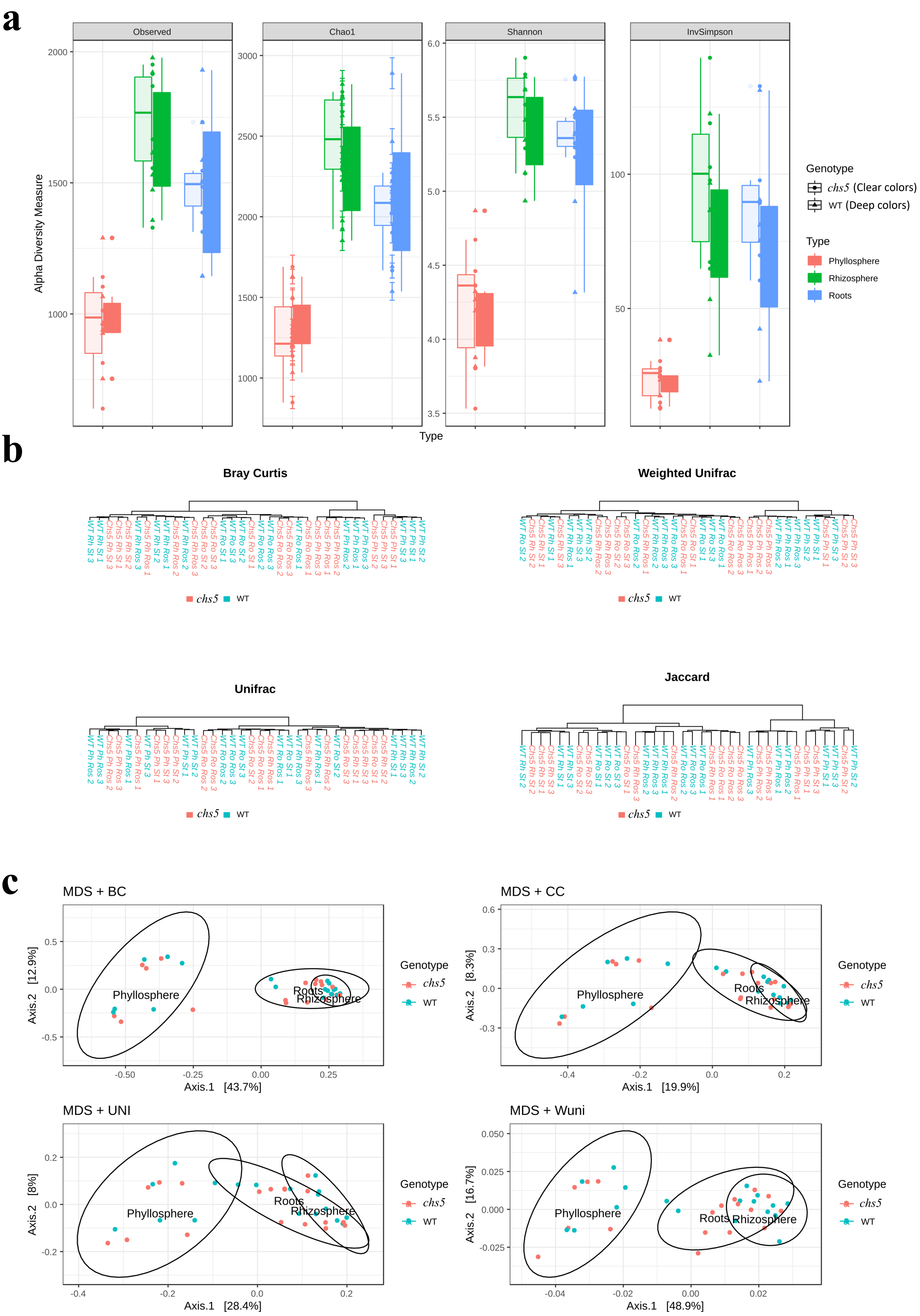

a
Observed
Chao1
Shannon
InvSimpson
Alpha Diversity Measure
Type
Genotype
chs5 (Clear colors)
WT (Deep colors)
Type
Phyllosphere
Rhizosphere
Roots
b
Bray Curtis
Weighted Unifrac
Unifrac
Jaccard
chs5
WT
c
MDS + BC
MDS + CC
MDS + UNI
MDS + Wuni
Phyllosphere
Roots
Rhizosphere
Axis.1 [43.7%]
Axis.2 [12.9%]
Axis.1 [19.9%]
Axis.2 [8.3%]
Axis.1 [28.4%]
Axis.2 [8%]
Axis.1 [48.9%]
Axis.2 [16.7%]
Genotype
chs5
WT

Figure S6

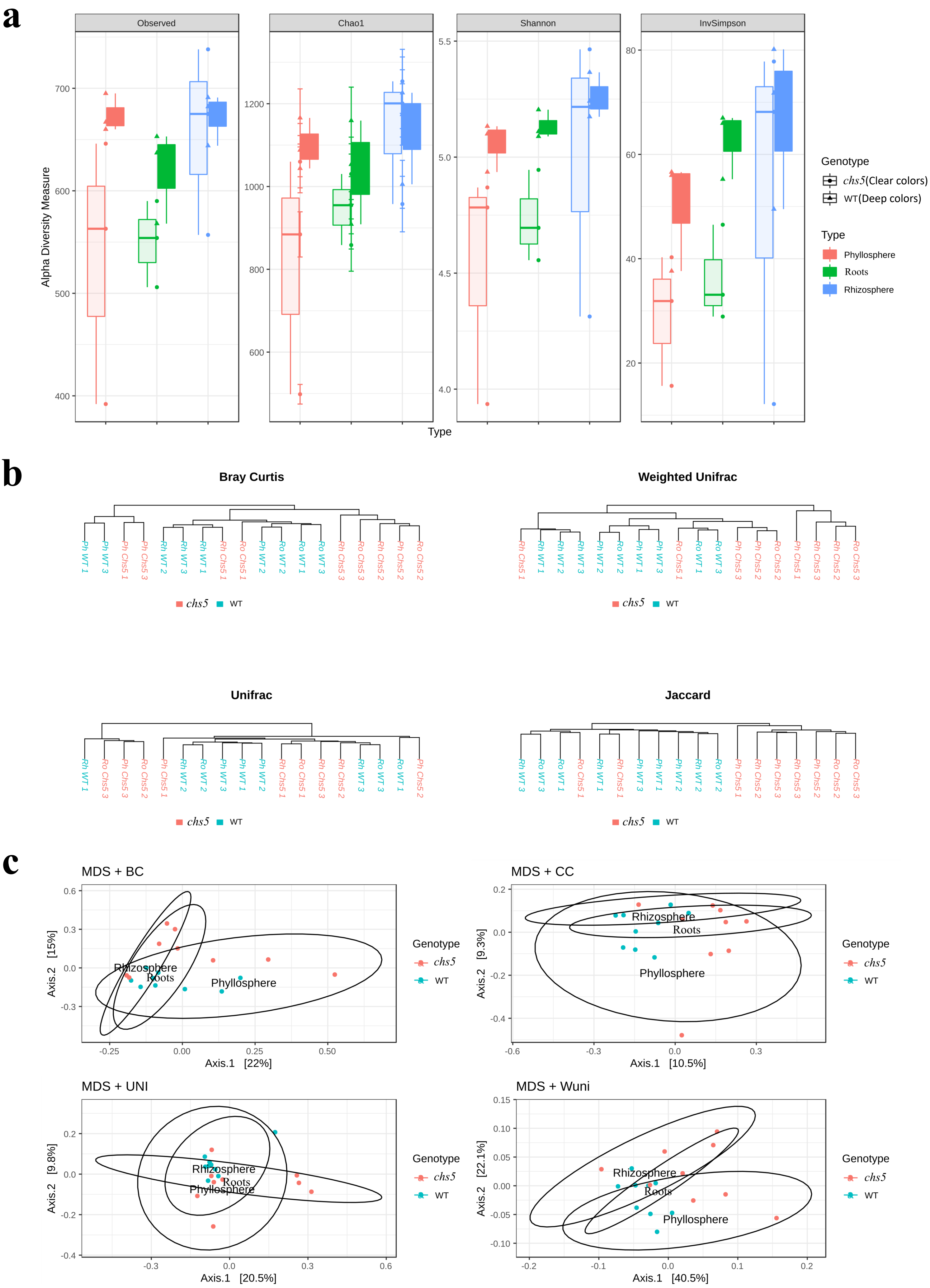
a
Observed
Chao1
Shannon
InvSimpson
Alpha Diversity Measure
Type
Genotype
chs5(Clear colors)
WT(Deep colors)
Type
Phyllosphere
Roots
Rhizosphere
b
Bray Curtis
Weighted Unifrac
Unifrac
Jaccard
chs5
WT
c
MDS + BC
MDS + CC
MDS + UNI
MDS + Wuni
Axis.1 [22%]
Axis.2 [15%]
Axis.1 [10.5%]
Axis.2 [9.3%]
Axis.1 [20.5%]
Axis.2 [9.8%]
Axis.1 [40.5%]
Axis.2 [22.1%]
Rhizosphere
Roots
Phyllosphere
Genotype
chs5
WT

Figure S7

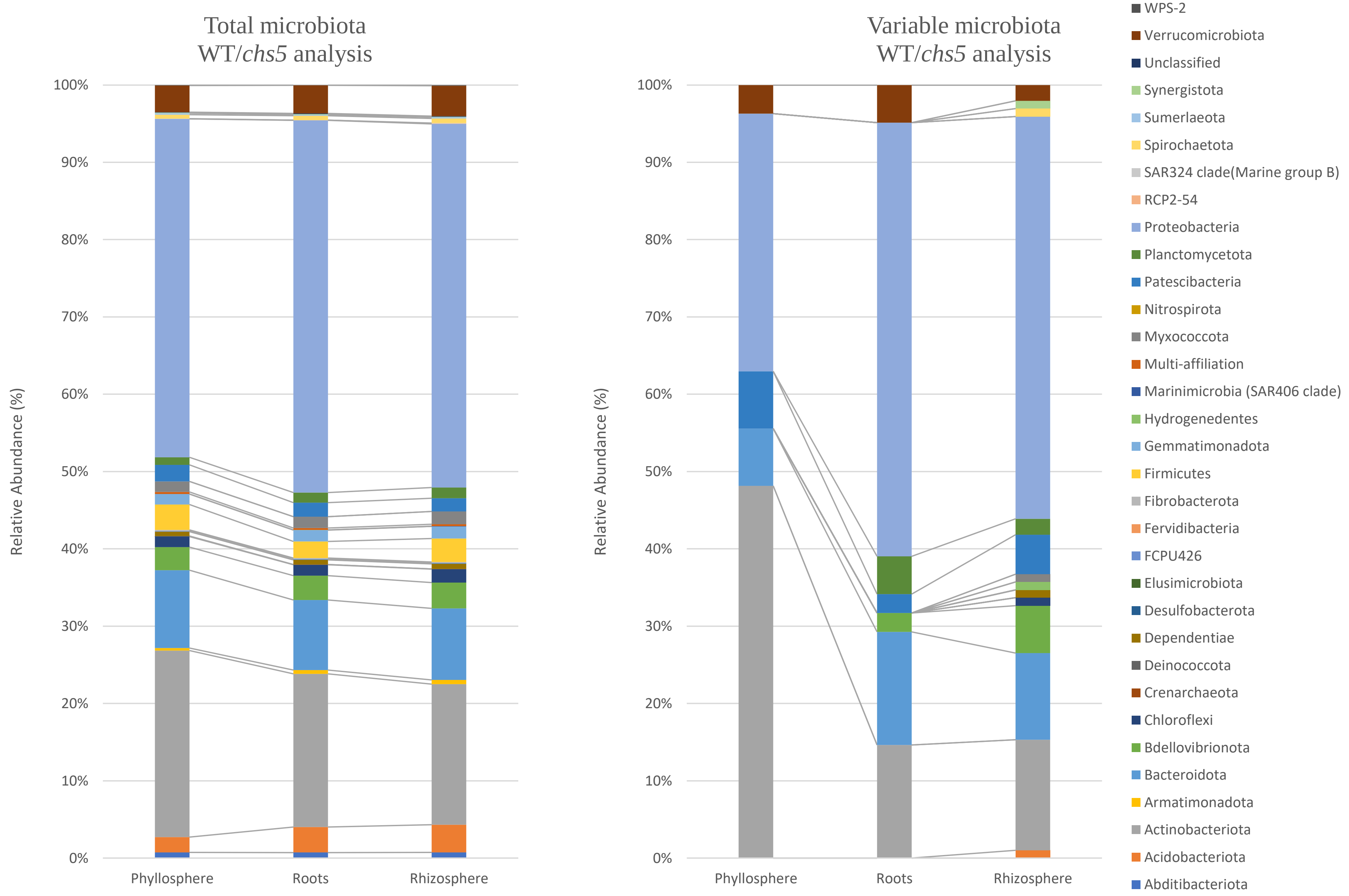

Total microbiota
WT/chs5 analysis
Variable microbiota
WT/chs5 analysis
Relative Abundance (%)
100%
90%
80%
70%
60%
50%
40%
30%
20%
10%
0%
Phyllosphere
Roots
Rhizosphere
WPS-2
Verrucomicrobiota
Unclassified
Synergistota
Sumerlaeota
Spirochaetota
SAR324 clade(Marine group B)
RCP2-54
Proteobacteria
Planctomycetota
Patescibacteria
Nitrospirota
Myxococcota
Multi-affiliation
Marinimicrobia (SAR406 clade)
Hydrogenedentes
Gemmatimonadota
Firmicutes
Fibrobacterota
Fervidibacteria
FCPU426
Elusimicrobiota
Desulfobacterota
Dependentiae
Deinococcota
Crenarchaeota
Chloroflexi
Bdellovibrionota
Bacteroidota
Armatimonadota
Actinobacteriota
Acidobacteriota
Abditibacteriota

Figure S8

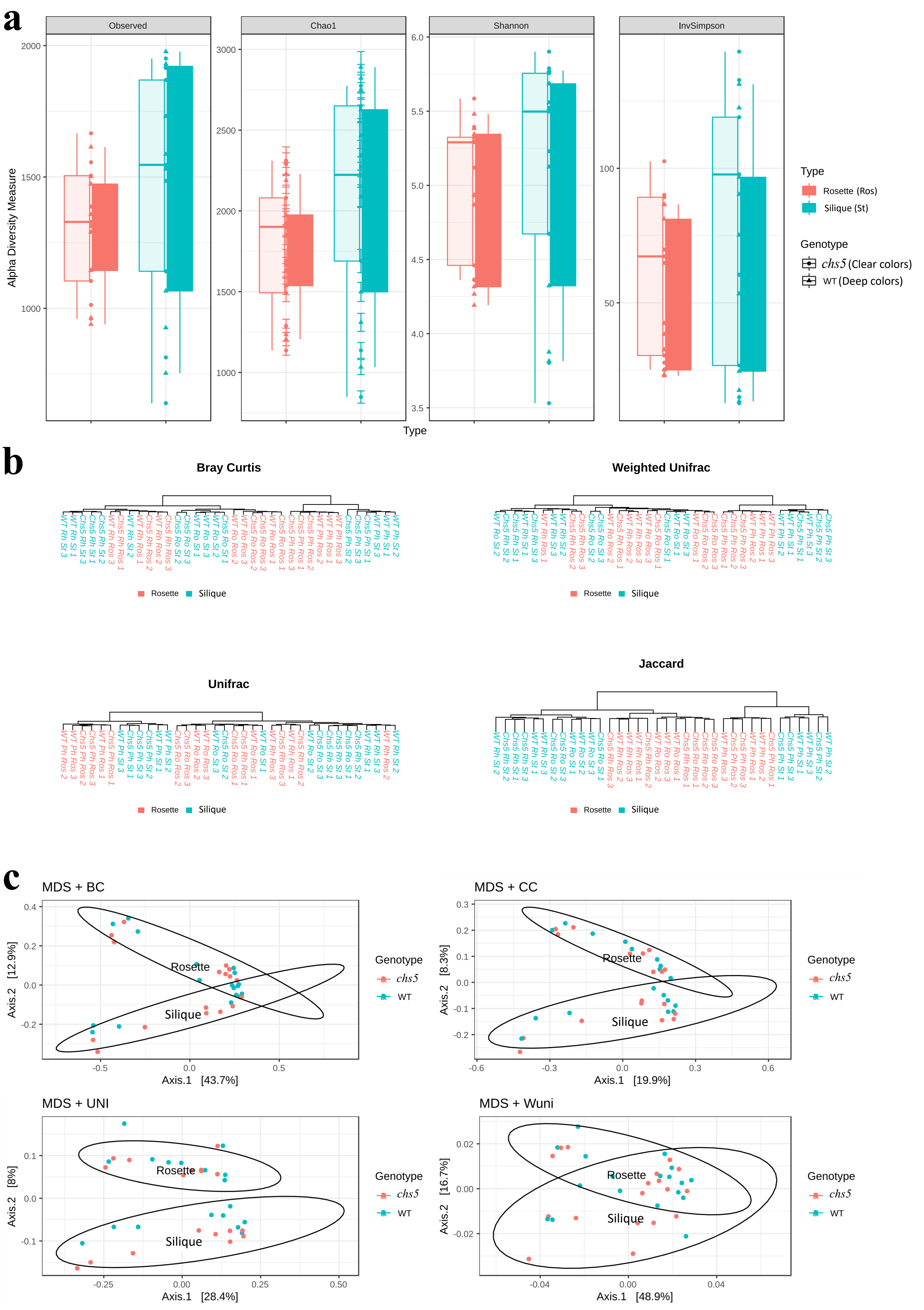

a
Observed
Chao1
Shannon
InvSimpson
Alpha Diversity Measure
Type
Rosette (Ros)
Silique (St)
Genotype
chs5 (Clear colors)
WT (Deep colors)
b
Bray Curtis
Weighted Unifrac
Unifrac
Jaccard
Rosette
Silique
c
MDS + BC
MDS + CC
MDS + UNI
MDS + Wuni
Axis.1 [43.7%]
Axis.2 [12.9%]
Axis.1 [19.9%]
Axis.2 [8.3%]
Axis.1 [28.4%]
Axis.2 [8%]
Axis.1 [48.9%]
Axis.2 [16.7%]
Rosette
Silique
Genotype
chs5
WT

Figure S9

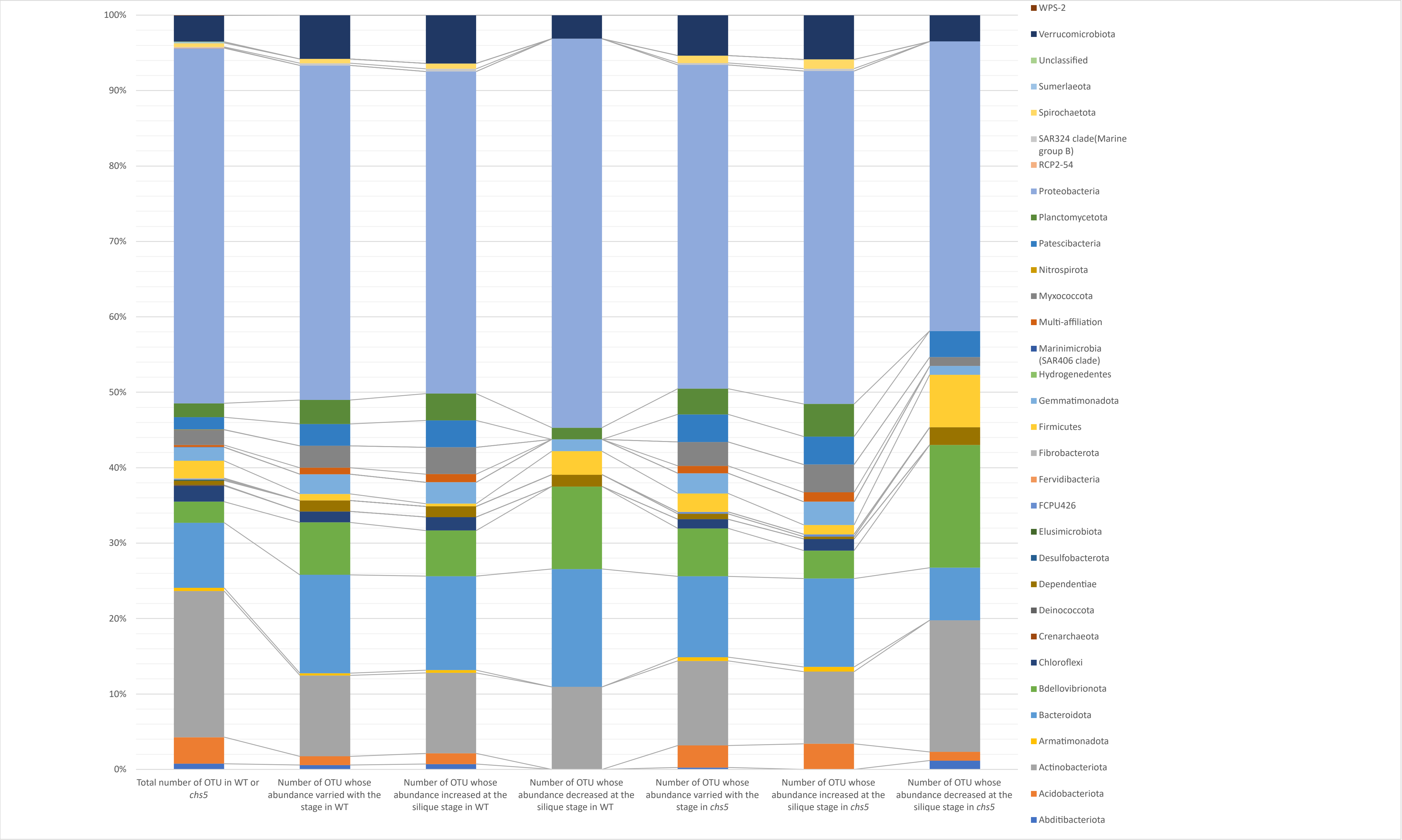
100%
90%
80%
70%
60%
50%
40%
30%
20%
10%
0%
Total number of OTU in WT or chs5
Number of OTU whose abundance varried with the stage in WT
Number of OTU whose abundance increased at the silique stage in WT
Number of OTU whose abundance decreased at the silique stage in WT
Number of OTU whose abundance varried with the stage in chs5
Number of OTU whose abundance increased at the silique stage in chs5
Number of OTU whose abundance decreased at the silique stage in chs5
WPS-2
Verrucomicrobiota
Unclassified
Sumerlaeota
Spirochaetota
SAR324 clade(Marine group B)
RCP2-54
Proteobacteria
Planctomycetota
Patescibacteria
Nitrospirota
Myxococcota
Multi-affiliation
Marinimicrobia (SAR406 clade)
Hydrogenedentes
Gemmatimonadota
Firmicutes
Fibrobacterota
Fervidibacteria
FCPU426
Elusimicrobiota
Desulfobacterota
Dependentiae
Deinococcota
Crenarchaeota
Chloroflexi
Bdellovibrionota
Bacteroidota
Armatimonadota
Actinobacteriota
Acidobacteriota
Abditibacteriota

Figure S10

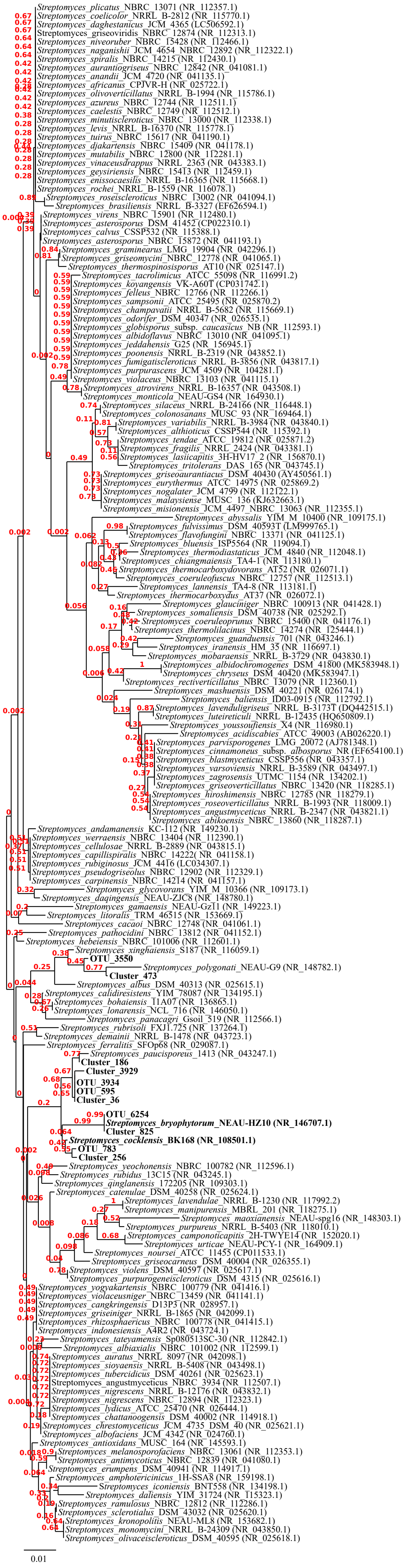

Figure S11

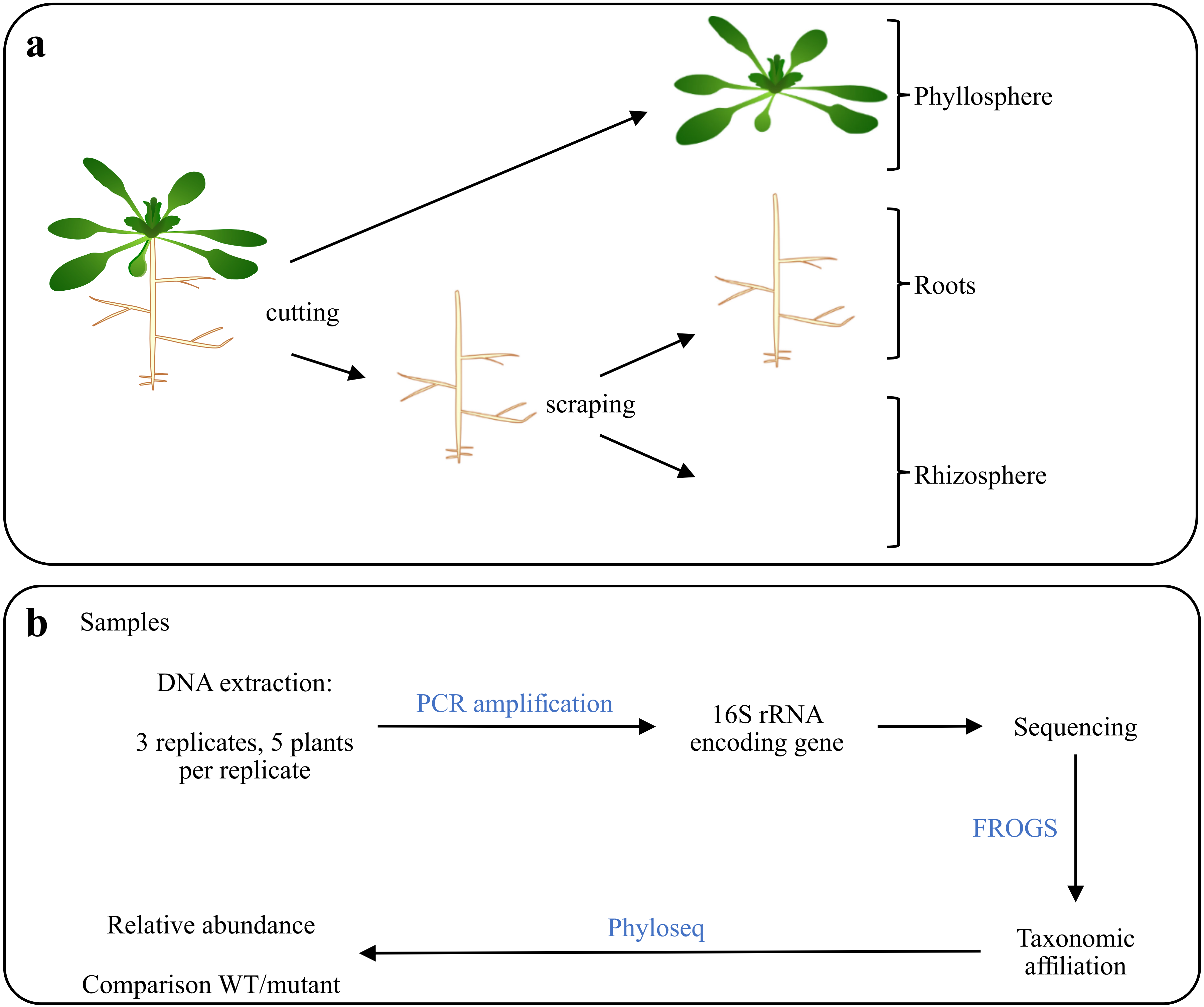
a
cutting
scraping
Phyllosphere
Roots
Rhizosphere
b
Samples
DNA extraction:
3 replicates, 5 plants per replicate
PCR amplification
16S rRNA encoding gene
Sequencing
FROGS
Taxonomic affiliation
Phyloseq
Relative abundance
Comparison WT/mutant

Figure S12

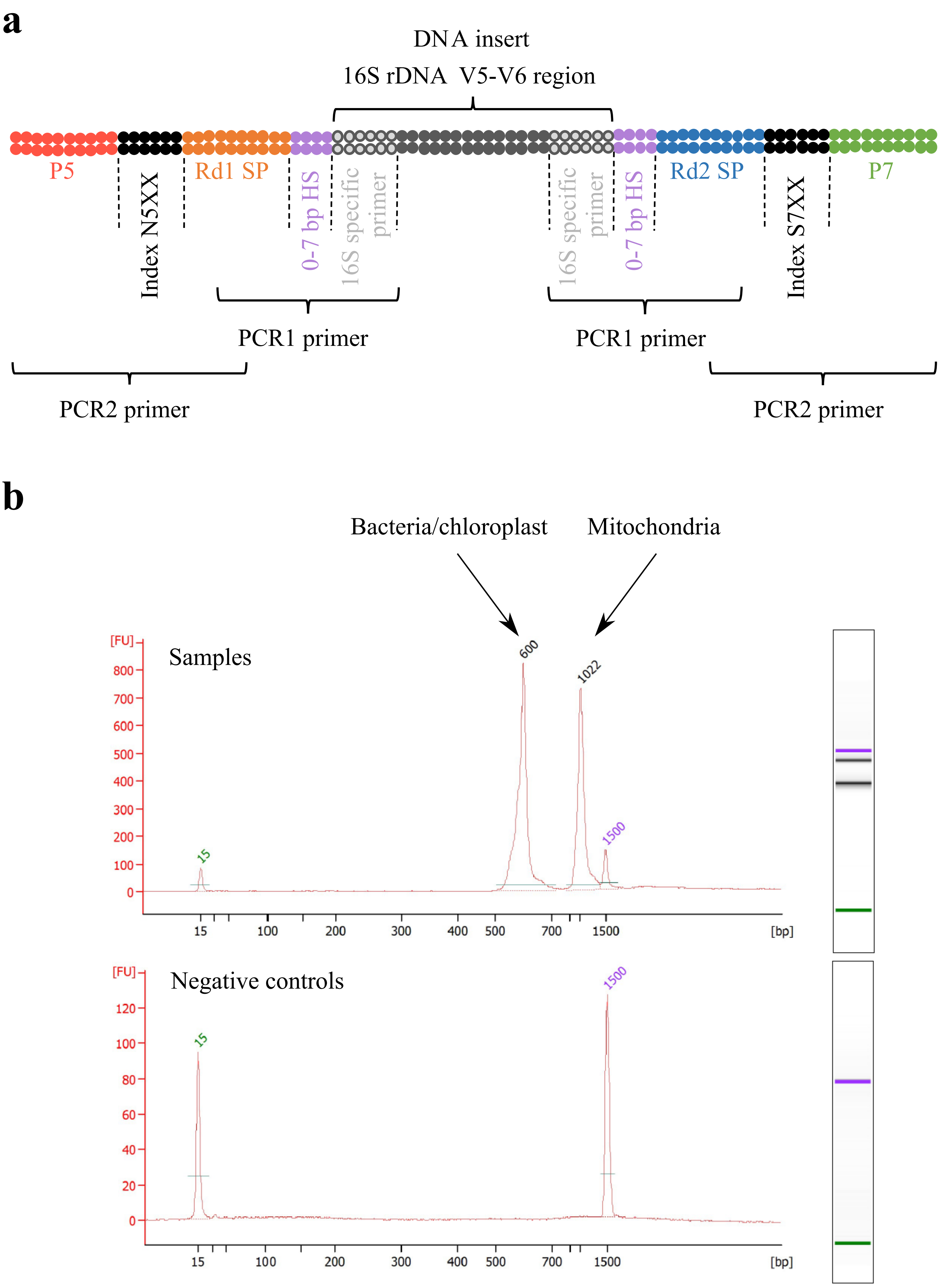
a
DNA insert
16S rDNA V5-V6 region
P5
Index N5XX
Rd1 SP
0-7 bp HS
16S specific primer
16S specific primer
0-7 bp HS
Rd2 SP
Index S7XX
P7
PCR1 primer
PCR1 primer
PCR2 primer
PCR2 primer
b
Bacteria/chloroplast
Mitochondria
Samples
[FU]
800
700
600
500
400
300
200
100
0
15
600
1022
1500
15
100
200
300
400
500
700
1500
[bp]
Negative controls
[FU]
120
100
80
60
40
20
0
15
1500
15
100
200
300
400
500
700
1500
[bp]

Figure S13

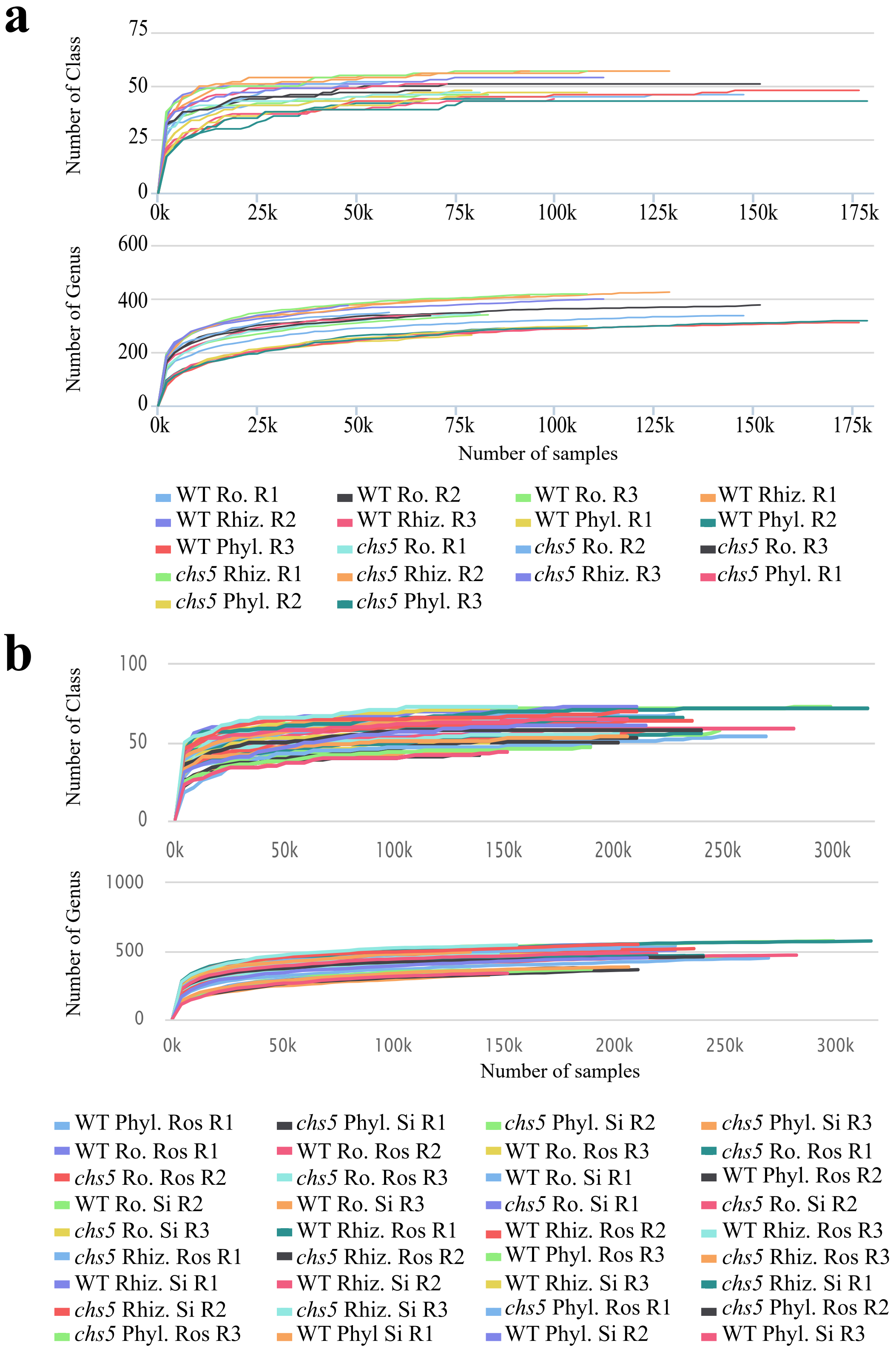
a
Number of Class
75
50
25
0
0k
25k
50k
75k
100k
125k
150k
175k
Number of Genus
600
400
200
0
0k
25k
50k
75k
100k
125k
150k
175k
Number of samples
WT Ro. R1
WT Ro. R2
WT Ro. R3
WT Rhiz. R1
WT Rhiz. R2
WT Rhiz. R3
WT Phyl. R1
WT Phyl. R2
WT Phyl. R3
chs5 Ro. R1
chs5 Ro. R2
chs5 Ro. R3
chs5 Rhiz. R1
chs5 Rhiz. R2
chs5 Rhiz. R3
chs5 Phyl. R1
chs5 Phyl. R2
chs5 Phyl. R3
b
Number of Class
100
50
0
0k
50k
100k
150k
200k
250k
300k
Number of Genus
1000
500
0
0k
50k
100k
150k
200k
250k
300k
Number of samples
WT Phyl. Ros R1
chs5 Phyl. Si R1
chs5 Phyl. Si R2
chs5 Phyl. Si R3
WT Ro. Ros R1
WT Ro. Ros R2
WT Ro. Ros R3
chs5 Ro. Ros R1
chs5 Ro. Ros R2
chs5 Ro. Ros R3
WT Ro. Si R1
WT Phyl. Ros R2
WT Ro. Si R2
WT Ro. Si R3
chs5 Ro. Si R1
chs5 Ro. Si R2
chs5 Ro. Si R3
WT Rhiz. Ros R1
WT Rhiz. Ros R2
WT Rhiz. Ros R3
chs5 Rhiz. Ros R1
chs5 Rhiz. Ros R2
WT Phyl. Ros R3
chs5 Rhiz. Ros R3
WT Rhiz. Si R1
WT Rhiz. Si R2
WT Rhiz. Si R3
chs5 Rhiz. Si R1
chs5 Rhiz. Si R2
chs5 Rhiz. Si R3
chs5 Phyl. Ros R1
chs5 Phyl. Ros R2
chs5 Phyl. Ros R3
WT Phyl Si R1
WT Phyl. Si R2
WT Phyl. Si R3

c

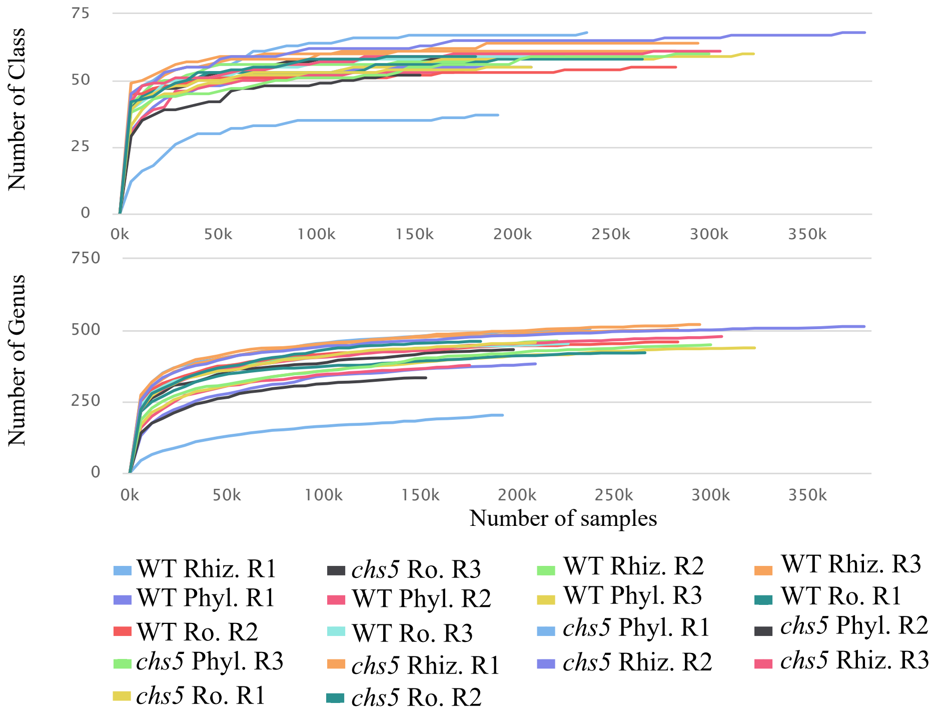
Number of Class
75
50
25
0
0k
50k
100k
150k
200k
250k
300k
350k
Number of Genus
750
500
250
0
Number of samples
WT Rhiz. R1
chs5 Ro. R3
WT Rhiz. R2
WT Rhiz. R3
WT Phyl. R1
WT Phyl. R2
WT Phyl. R3
WT Ro. R1
WT Ro. R2
WT Ro. R3
chs5 Phyl. R1
chs5 Phyl. R2
chs5 Phyl. R3
chs5 Rhiz. R1
chs5 Rhiz. R2
chs5 Rhiz. R3
chs5 Ro. R1
chs5 Ro. R2
